# 3-Anilino-1-ferrocenylpropan-1-one

**DOI:** 10.1107/S1600536812003492

**Published:** 2012-01-31

**Authors:** Zorica Leka, Sladjana B. Novaković, Dragana Stevanović, Goran A. Bogdanović, Rastko D. Vukićević

**Affiliations:** a Faculty of Metallurgy and Technology, University of Montenegro, Cetinjski put bb, 81000 Podgorica, Montenegro; b ’Vinča’ Institute of Nuclear Sciences, Laboratory of Theoretical Physics and Condensed Matter Physics, PO Box 522, 11001 Belgrade, Serbia; c Department of Chemistry, Faculty of Science, University of Kragujevac, R. Domanovića 12, 34000 Kragujevac, Serbia

## Abstract

In the title ferrocene derivative, [Fe(C_5_H_5_)(C_14_H_14_NO)], the dihedral angle between the mean planes of the phenyl ring and the substituted cyclo­penta­dienyl ring is 84.4 (1)°. The mol­ecules are connected into centrosymmetric dimers *via* N—H⋯O hydrogen bonds. In addition, C—H⋯O and C—H⋯N contacts stabilize the crystal packing.

## Related literature

For the physico-chemical properties of ferrocene-based compounds, see: Togni & Hayashi (1995 ▶). For related crystal structures and details of the synthesis, see: Damljanović *et al.* (2011 ▶); Stevanović *et al.* (2012 ▶); Leka, Novaković, Stevanović *et al.* (2012 ▶); Leka, Novaković, Pejović *et al.* (2012 ▶).
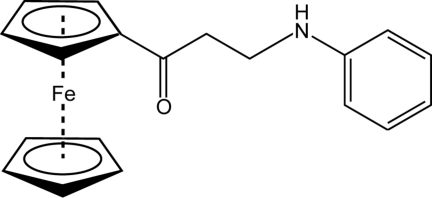


## Experimental

### 

#### Crystal data

[Fe(C_5_H_5_)(C_14_H_14_NO)]
*M* *_r_* = 333.20
Triclinic, 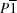

*a* = 7.605 (3) Å
*b* = 9.748 (3) Å
*c* = 12.098 (4) Å
α = 86.036 (4)°
β = 73.869 (4)°
γ = 68.684 (3)°
*V* = 802.1 (5) Å^3^
*Z* = 2
Mo *K*α radiation
μ = 0.94 mm^−1^
*T* = 293 K
0.26 × 0.25 × 0.18 mm

#### Data collection

Enraf–Nonius CAD-4 diffractometer
3395 measured reflections
3143 independent reflections
2449 reflections with *I* > 2σ(*I*)
*R*_int_ = 0.017
3 standard reflections every 60 min intensity decay: none

#### Refinement

*R*[*F* ^2^ > 2σ(*F* ^2^)] = 0.034
*wR*(*F* ^2^) = 0.091
*S* = 1.05
3143 reflections
203 parameters
H atoms treated by a mixture of independent and constrained refinement
Δρ_max_ = 0.21 e Å^−3^
Δρ_min_ = −0.21 e Å^−3^

### 

Data collection: *CAD-4 Software* (Enraf–Nonius, 1989 ▶); cell refinement: *CAD-4 Software*; data reduction: *CAD-4 Software*; program(s) used to solve structure: *SHELXS97* (Sheldrick, 2008 ▶); program(s) used to refine structure: *SHELXL97* (Sheldrick, 2008 ▶); molecular graphics: *ORTEP-3* (Farrugia, 1997 ▶) and *POV-RAY* (Persistence of Vision, 2004 ▶); software used to prepare material for publication: *WinGX* (Farrugia, 1999 ▶), *PLATON* (Spek, 2009 ▶) and *PARST* (Nardelli, 1995 ▶).

## Supplementary Material

Crystal structure: contains datablock(s) I, global. DOI: 10.1107/S1600536812003492/bt5790sup1.cif

Structure factors: contains datablock(s) I. DOI: 10.1107/S1600536812003492/bt5790Isup2.hkl

Additional supplementary materials:  crystallographic information; 3D view; checkCIF report

## Figures and Tables

**Table 1:** Hydrogen-bond geometry (Å, °)

*D*—H⋯*A*	*D*—H	H⋯*A*	*D*⋯*A*	*D*—H⋯*A*
N1—H1N⋯O1^i^	0.80 (3)	2.30 (3)	3.082 (3)	164 (2)
C19—H19⋯O1^i^	0.93	2.65	3.425 (3)	141
C4—H4⋯N1^ii^	0.93	2.63	3.489 (3)	153

Symmetry codes: (i) 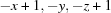; (ii) 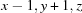.

**Fig. 1.**
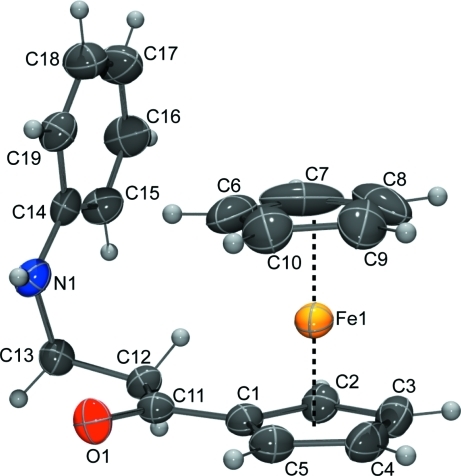
The molecular structure of the title compound, with atom labels and 40% probability displacement ellipsoids for non-H atoms.

**Fig. 2.**
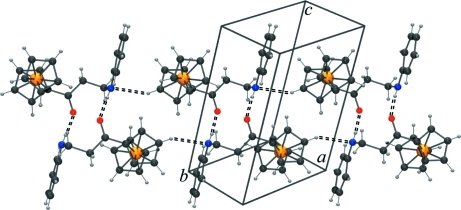
Part of the crystal packing showing the interconnection of dimers into a chain.

## supplementary crystallographic information

### Comment

Derivatives of ferrocene have attracted great interest due to their physical, chemical and biological properties. The ease of functionalization of the ferrocene (Fc) unit led to structurally diverse compounds with numerous applications. In that context Mannich bases (Mannich ketones; β-aminoketones) containing a Fc unit might be very useful synthetic components as they can be converted into a range of other derivatives, such as 1,3-aminoalcohols. Here we report the crystal structure of the Mannich base, 1-Ferrocenyl-3-(phenylamino)propan-1- one (I), synthesized according to the previously reported procedure (Damljanović *et al.*, 2011).

In the title compound (Figure 1) the cyclopentadienyl rings (Cp) within the Fc unit take an almost eclipsed geometry, where the smallest C—C*g*1—C*g*2—C torsion angle has the value of 5.1° (*Cg*1 and *Cg*2 are centroids of the corresponding Cp rings). Bond lengths of the Fc unit have the expected values and the Cp rings show only a small mutual tilting of 1.5 (2)°. The distances of Fe1 to the centroids of the two Cp rings are 1.65 and 1.66 Å, respectively. The C1—O1 carbonyl group lies approximately in the plane of the substituted Cp1 ring with the O1—C11—C1—C5 torsion angle of 3.9 (3)°. Similarly, the atoms of phenylamino moiety are approximately co-planar as evidenced from the N1—C14—C15—C16 torsion angle of 175.5 (2)°. The torsion angle C11—C12—C13—N1 of 72.3 (2)°, on the other hand, indicates a significant twisting between two aromatic parts of the molecule, eventually the phenylamino moiety takes an almost orthogonal position with respect to the substituted Cp ring. The dihedral angle between the best planes of the two rings, phenyl and substituted Cp is 84.4 (1)°.

Molecules are organized into centrosymmetric dimers *via* the N—H···O and C19—H19···O1 hydrogen bonds. These dimers further arrange into the chain trough the C4—H4···N1 interaction (Figure 2).

### Experimental

The compound was obtained by an aza-Michael addition of the corresponding arylamine to acryloylferrocene. The reaction was performed by microwave (MW) irradiation (500 W/5 min) of a mixture of reactants and montmorillonite K-10, without a solvent as described by Damljanović *et al.* (2011).

### Refinement

H atoms bonded to C atoms were placed at geometrically calculated positions and refined using a riding model. C—H distances were fixed to 0.93 and 0.97 Å from aromatic and methylene C atoms, respectively. The *U*_iso_(H) values were equal to 1.2 times *U*_eq_ of the corresponding parent atom. H atom attached to N atom was isotropically refined.

### Crystal data

**Table:**

[Fe(C_5_H_5_)(C_14_H_14_NO)]	*Z* = 2
*M**_r_* = 333.20	*F*(000) = 348
Triclinic, *P*1	*D*_x_ = 1.380 Mg m^−^^3^
Hall symbol: -P 1	Mo *K*α radiation, λ = 0.71073 Å
*a* = 7.605 (3) Å	Cell parameters from 25 reflections
*b* = 9.748 (3) Å	θ = 10.3–15.4°
*c* = 12.098 (4) Å	µ = 0.94 mm^−^^1^
α = 86.036 (4)°	*T* = 293 K
β = 73.869 (4)°	Prismatic, orange
γ = 68.684 (3)°	0.26 × 0.25 × 0.18 mm
*V* = 802.1 (5) Å^3^	

### Data collection

**Table:**

Enraf–Nonius CAD-4 diffractometer	*R*_int_ = 0.017
Radiation source: fine-focus sealed tube	θ_max_ = 26.0°, θ_min_ = 1.8°
graphite	*h* = 0→9
ω/2θ scans	*k* = −11→11
3395 measured reflections	*l* = −14→14
3143 independent reflections	3 standard reflections every 60 min
2449 reflections with *I* > 2σ(*I*)	intensity decay: none

### Refinement

**Table:**

Refinement on *F*^2^	Primary atom site location: structure-invariant direct methods
Least-squares matrix: full	Secondary atom site location: difference Fourier map
*R*[*F*^2^ > 2σ(*F*^2^)] = 0.034	Hydrogen site location: inferred from neighbouring sites
*wR*(*F*^2^) = 0.091	H atoms treated by a mixture of independent and constrained refinement
*S* = 1.05	*w* = 1/[σ^2^(*F*_o_^2^) + (0.0483*P*)^2^ + 0.0454*P*] where *P* = (*F*_o_^2^ + 2*F*_c_^2^)/3
3143 reflections	(Δ/σ)_max_ = 0.001
203 parameters	Δρ_max_ = 0.21 e Å^−^^3^
0 restraints	Δρ_min_ = −0.20 e Å^−^^3^

### Fractional atomic coordinates and isotropic or equivalent isotropic displacement parameters (Å^2^)

**Table:**

	*x*	*y*	*z*	*U*_iso_*/*U*_eq_	
Fe01	−0.00076 (5)	0.31825 (3)	0.71233 (3)	0.04475 (12)	
O1	0.2742 (2)	0.01729 (17)	0.48442 (13)	0.0510 (4)	
N1	0.4975 (3)	−0.2119 (2)	0.64319 (17)	0.0458 (4)	
C1	−0.0181 (3)	0.1691 (2)	0.61151 (18)	0.0394 (5)	
C2	−0.1759 (3)	0.2006 (2)	0.7152 (2)	0.0461 (5)	
H2	−0.2000	0.1326	0.7695	0.055*	
C3	−0.2883 (3)	0.3525 (3)	0.7207 (2)	0.0546 (6)	
H3	−0.3992	0.4020	0.7792	0.065*	
C4	−0.2033 (4)	0.4165 (3)	0.6219 (2)	0.0569 (6)	
H4	−0.2493	0.5155	0.6046	0.068*	
C5	−0.0372 (4)	0.3057 (2)	0.5537 (2)	0.0480 (5)	
H5	0.0449	0.3187	0.4841	0.058*	
C6	0.2740 (5)	0.2429 (4)	0.7347 (4)	0.0846 (10)	
H6	0.3757	0.1568	0.7017	0.102*	
C7	0.1301 (8)	0.2555 (5)	0.8412 (4)	0.1119 (16)	
H7	0.1189	0.1796	0.8904	0.134*	
C8	0.0069 (6)	0.4060 (5)	0.8582 (3)	0.0975 (13)	
H8	−0.0997	0.4477	0.9214	0.117*	
C9	0.0746 (5)	0.4800 (4)	0.7630 (3)	0.0807 (9)	
H9	0.0199	0.5801	0.7512	0.097*	
C10	0.2371 (4)	0.3796 (4)	0.6889 (3)	0.0771 (9)	
H10	0.3096	0.4016	0.6191	0.093*	
C11	0.1447 (3)	0.0293 (2)	0.57366 (17)	0.0373 (4)	
C12	0.1448 (3)	−0.1032 (2)	0.64668 (18)	0.0402 (5)	
H12B	0.0398	−0.1334	0.6402	0.048*	
H12A	0.1188	−0.0752	0.7267	0.048*	
C13	0.3384 (3)	−0.2331 (2)	0.61176 (19)	0.0451 (5)	
H13A	0.3196	−0.3206	0.6478	0.054*	
H13B	0.3752	−0.2501	0.5290	0.054*	
C14	0.5038 (3)	−0.2122 (2)	0.75672 (19)	0.0414 (5)	
C15	0.3963 (4)	−0.2745 (3)	0.8440 (2)	0.0554 (6)	
H15	0.3092	−0.3121	0.8283	0.066*	
C16	0.4193 (4)	−0.2805 (4)	0.9544 (2)	0.0714 (8)	
H16	0.3476	−0.3232	1.0116	0.086*	
C17	0.5448 (5)	−0.2254 (4)	0.9813 (2)	0.0772 (9)	
H17	0.5602	−0.2317	1.0553	0.093*	
C18	0.6488 (4)	−0.1596 (3)	0.8953 (2)	0.0681 (7)	
H18	0.7327	−0.1197	0.9122	0.082*	
C19	0.6284 (3)	−0.1532 (3)	0.7854 (2)	0.0519 (6)	
H19	0.6989	−0.1088	0.7291	0.062*	
H1N	0.546 (3)	−0.161 (3)	0.600 (2)	0.044 (7)*	

### Atomic displacement parameters (Å^2^)

**Table:**

	*U*^11^	*U*^22^	*U*^33^	*U*^12^	*U*^13^	*U*^23^
Fe01	0.04078 (19)	0.04572 (19)	0.0522 (2)	−0.01795 (14)	−0.01521 (14)	−0.00289 (13)
O1	0.0527 (10)	0.0508 (9)	0.0448 (9)	−0.0228 (8)	−0.0013 (7)	0.0055 (7)
N1	0.0391 (10)	0.0494 (11)	0.0490 (11)	−0.0190 (9)	−0.0103 (9)	0.0123 (9)
C1	0.0382 (11)	0.0417 (11)	0.0448 (11)	−0.0191 (9)	−0.0152 (9)	0.0021 (9)
C2	0.0380 (11)	0.0497 (13)	0.0538 (13)	−0.0216 (10)	−0.0096 (10)	0.0024 (10)
C3	0.0386 (12)	0.0520 (13)	0.0711 (16)	−0.0138 (11)	−0.0136 (11)	−0.0048 (12)
C4	0.0558 (15)	0.0407 (12)	0.0758 (17)	−0.0088 (11)	−0.0326 (13)	0.0045 (11)
C5	0.0562 (14)	0.0460 (12)	0.0485 (12)	−0.0222 (11)	−0.0214 (11)	0.0097 (10)
C6	0.0646 (19)	0.077 (2)	0.124 (3)	−0.0120 (16)	−0.056 (2)	−0.018 (2)
C7	0.167 (4)	0.134 (4)	0.114 (3)	−0.103 (4)	−0.109 (3)	0.056 (3)
C8	0.095 (3)	0.155 (4)	0.068 (2)	−0.074 (3)	−0.0120 (18)	−0.038 (2)
C9	0.0660 (19)	0.0687 (18)	0.117 (3)	−0.0305 (16)	−0.0230 (18)	−0.0293 (18)
C10	0.0559 (17)	0.091 (2)	0.096 (2)	−0.0390 (17)	−0.0160 (16)	−0.0179 (18)
C11	0.0383 (11)	0.0425 (11)	0.0383 (11)	−0.0211 (9)	−0.0133 (9)	0.0044 (8)
C12	0.0367 (11)	0.0430 (11)	0.0446 (11)	−0.0193 (9)	−0.0110 (9)	0.0064 (9)
C13	0.0473 (13)	0.0392 (11)	0.0501 (12)	−0.0178 (10)	−0.0124 (10)	0.0022 (9)
C14	0.0320 (10)	0.0373 (10)	0.0487 (12)	−0.0079 (9)	−0.0080 (9)	0.0049 (9)
C15	0.0509 (14)	0.0659 (15)	0.0574 (14)	−0.0319 (12)	−0.0152 (11)	0.0138 (12)
C16	0.0685 (19)	0.100 (2)	0.0539 (15)	−0.0450 (17)	−0.0138 (13)	0.0213 (15)
C17	0.074 (2)	0.111 (3)	0.0522 (15)	−0.0374 (18)	−0.0210 (14)	0.0063 (16)
C18	0.0594 (17)	0.086 (2)	0.0691 (17)	−0.0334 (15)	−0.0214 (14)	−0.0047 (15)
C19	0.0422 (13)	0.0558 (14)	0.0596 (14)	−0.0232 (11)	−0.0099 (11)	0.0051 (11)

### Geometric parameters (Å, °)

**Table:**

Fe01—C7	2.021 (3)	C6—H6	0.9300
Fe01—C1	2.023 (2)	C7—C8	1.417 (6)
Fe01—C5	2.031 (2)	C7—H7	0.9300
Fe01—C6	2.037 (3)	C8—C9	1.392 (5)
Fe01—C8	2.038 (3)	C8—H8	0.9300
Fe01—C2	2.042 (2)	C9—C10	1.383 (4)
Fe01—C9	2.043 (3)	C9—H9	0.9300
Fe01—C10	2.046 (3)	C10—H10	0.9300
Fe01—C4	2.051 (2)	C11—C12	1.513 (3)
Fe01—C3	2.063 (3)	C12—C13	1.524 (3)
O1—C11	1.221 (2)	C12—H12B	0.9700
N1—C14	1.387 (3)	C12—H12A	0.9700
N1—C13	1.449 (3)	C13—H13A	0.9700
N1—H1N	0.80 (2)	C13—H13B	0.9700
C1—C2	1.434 (3)	C14—C15	1.394 (3)
C1—C5	1.439 (3)	C14—C19	1.399 (3)
C1—C11	1.464 (3)	C15—C16	1.390 (4)
C2—C3	1.410 (3)	C15—H15	0.9300
C2—H2	0.9300	C16—C17	1.369 (4)
C3—C4	1.413 (4)	C16—H16	0.9300
C3—H3	0.9300	C17—C18	1.393 (4)
C4—C5	1.413 (3)	C17—H17	0.9300
C4—H4	0.9300	C18—C19	1.375 (4)
C5—H5	0.9300	C18—H18	0.9300
C6—C10	1.369 (5)	C19—H19	0.9300
C6—C7	1.420 (6)		
			
C7—Fe01—C1	121.56 (15)	Fe01—C4—H4	126.7
C7—Fe01—C5	156.29 (18)	C4—C5—C1	107.5 (2)
C1—Fe01—C5	41.58 (9)	C4—C5—Fe01	70.49 (14)
C7—Fe01—C6	40.95 (17)	C1—C5—Fe01	68.90 (12)
C1—Fe01—C6	108.24 (11)	C4—C5—H5	126.2
C5—Fe01—C6	119.96 (13)	C1—C5—H5	126.2
C7—Fe01—C8	40.86 (17)	Fe01—C5—H5	125.9
C1—Fe01—C8	157.53 (15)	C10—C6—C7	108.1 (3)
C5—Fe01—C8	160.08 (16)	C10—C6—Fe01	70.77 (17)
C6—Fe01—C8	67.93 (15)	C7—C6—Fe01	68.91 (19)
C7—Fe01—C2	109.56 (13)	C10—C6—H6	126.0
C1—Fe01—C2	41.32 (9)	C7—C6—H6	126.0
C5—Fe01—C2	69.15 (9)	Fe01—C6—H6	125.9
C6—Fe01—C2	127.82 (13)	C8—C7—C6	106.8 (3)
C8—Fe01—C2	122.33 (13)	C8—C7—Fe01	70.2 (2)
C7—Fe01—C9	67.70 (16)	C6—C7—Fe01	70.15 (19)
C1—Fe01—C9	160.84 (12)	C8—C7—H7	126.6
C5—Fe01—C9	123.30 (13)	C6—C7—H7	126.6
C6—Fe01—C9	66.68 (13)	Fe01—C7—H7	124.7
C8—Fe01—C9	39.90 (15)	C9—C8—C7	107.4 (3)
C2—Fe01—C9	156.23 (12)	C9—C8—Fe01	70.27 (17)
C7—Fe01—C10	67.42 (16)	C7—C8—Fe01	68.94 (18)
C1—Fe01—C10	124.95 (11)	C9—C8—H8	126.3
C5—Fe01—C10	106.64 (12)	C7—C8—H8	126.3
C6—Fe01—C10	39.17 (14)	Fe01—C8—H8	126.1
C8—Fe01—C10	66.99 (14)	C10—C9—C8	108.6 (3)
C2—Fe01—C10	163.30 (11)	C10—C9—Fe01	70.35 (16)
C9—Fe01—C10	39.54 (12)	C8—C9—Fe01	69.84 (17)
C7—Fe01—C4	162.65 (19)	C10—C9—H9	125.7
C1—Fe01—C4	68.75 (9)	C8—C9—H9	125.7
C5—Fe01—C4	40.49 (10)	Fe01—C9—H9	125.7
C6—Fe01—C4	154.06 (15)	C6—C10—C9	109.2 (3)
C8—Fe01—C4	124.72 (16)	C6—C10—Fe01	70.06 (18)
C2—Fe01—C4	67.94 (10)	C9—C10—Fe01	70.11 (17)
C9—Fe01—C4	107.24 (13)	C6—C10—H10	125.4
C10—Fe01—C4	120.04 (14)	C9—C10—H10	125.4
C7—Fe01—C3	126.91 (17)	Fe01—C10—H10	126.0
C1—Fe01—C3	68.67 (9)	O1—C11—C1	121.74 (18)
C5—Fe01—C3	68.33 (10)	O1—C11—C12	120.34 (18)
C6—Fe01—C3	164.78 (15)	C1—C11—C12	117.89 (17)
C8—Fe01—C3	108.87 (13)	C11—C12—C13	112.81 (17)
C2—Fe01—C3	40.18 (9)	C11—C12—H12B	109.0
C9—Fe01—C3	121.15 (12)	C13—C12—H12B	109.0
C10—Fe01—C3	154.80 (13)	C11—C12—H12A	109.0
C4—Fe01—C3	40.17 (10)	C13—C12—H12A	109.0
C14—N1—C13	122.08 (19)	H12B—C12—H12A	107.8
C14—N1—H1N	117.2 (17)	N1—C13—C12	113.66 (18)
C13—N1—H1N	113.7 (17)	N1—C13—H13A	108.8
C2—C1—C5	107.12 (19)	C12—C13—H13A	108.8
C2—C1—C11	127.89 (19)	N1—C13—H13B	108.8
C5—C1—C11	124.78 (19)	C12—C13—H13B	108.8
C2—C1—Fe01	70.05 (12)	H13A—C13—H13B	107.7
C5—C1—Fe01	69.52 (12)	N1—C14—C15	122.8 (2)
C11—C1—Fe01	121.47 (14)	N1—C14—C19	119.3 (2)
C3—C2—C1	108.2 (2)	C15—C14—C19	117.8 (2)
C3—C2—Fe01	70.69 (14)	C16—C15—C14	120.0 (2)
C1—C2—Fe01	68.63 (12)	C16—C15—H15	120.0
C3—C2—H2	125.9	C14—C15—H15	120.0
C1—C2—H2	125.9	C17—C16—C15	121.8 (3)
Fe01—C2—H2	126.4	C17—C16—H16	119.1
C2—C3—C4	108.2 (2)	C15—C16—H16	119.1
C2—C3—Fe01	69.13 (13)	C16—C17—C18	118.5 (3)
C4—C3—Fe01	69.46 (14)	C16—C17—H17	120.8
C2—C3—H3	125.9	C18—C17—H17	120.8
C4—C3—H3	125.9	C19—C18—C17	120.5 (3)
Fe01—C3—H3	127.1	C19—C18—H18	119.7
C5—C4—C3	108.9 (2)	C17—C18—H18	119.7
C5—C4—Fe01	69.02 (13)	C18—C19—C14	121.3 (2)
C3—C4—Fe01	70.36 (14)	C18—C19—H19	119.3
C5—C4—H4	125.5	C14—C19—H19	119.3
C3—C4—H4	125.5		
			
C7—Fe01—C1—C2	84.1 (2)	C2—Fe01—C6—C10	165.22 (17)
C5—Fe01—C1—C2	−118.02 (18)	C9—Fe01—C6—C10	−36.8 (2)
C6—Fe01—C1—C2	127.09 (18)	C4—Fe01—C6—C10	44.6 (3)
C8—Fe01—C1—C2	50.9 (4)	C3—Fe01—C6—C10	−161.0 (4)
C9—Fe01—C1—C2	−161.7 (3)	C1—Fe01—C6—C7	−117.5 (2)
C10—Fe01—C1—C2	167.13 (16)	C5—Fe01—C6—C7	−161.5 (2)
C4—Fe01—C1—C2	−80.32 (15)	C8—Fe01—C6—C7	38.9 (2)
C3—Fe01—C1—C2	−37.06 (14)	C2—Fe01—C6—C7	−75.6 (3)
C7—Fe01—C1—C5	−157.9 (2)	C9—Fe01—C6—C7	82.3 (2)
C6—Fe01—C1—C5	−114.89 (18)	C10—Fe01—C6—C7	119.1 (3)
C8—Fe01—C1—C5	168.9 (3)	C4—Fe01—C6—C7	163.8 (3)
C2—Fe01—C1—C5	118.02 (18)	C3—Fe01—C6—C7	−41.8 (5)
C9—Fe01—C1—C5	−43.7 (4)	C10—C6—C7—C8	−0.8 (3)
C10—Fe01—C1—C5	−74.85 (19)	Fe01—C6—C7—C8	−61.0 (2)
C4—Fe01—C1—C5	37.70 (14)	C10—C6—C7—Fe01	60.2 (2)
C3—Fe01—C1—C5	80.96 (15)	C1—Fe01—C7—C8	−161.36 (19)
C7—Fe01—C1—C11	−38.9 (3)	C5—Fe01—C7—C8	160.3 (3)
C5—Fe01—C1—C11	119.0 (2)	C6—Fe01—C7—C8	117.1 (3)
C6—Fe01—C1—C11	4.1 (2)	C2—Fe01—C7—C8	−117.2 (2)
C8—Fe01—C1—C11	−72.1 (4)	C9—Fe01—C7—C8	37.5 (2)
C2—Fe01—C1—C11	−122.9 (2)	C10—Fe01—C7—C8	80.4 (2)
C9—Fe01—C1—C11	75.4 (4)	C4—Fe01—C7—C8	−38.7 (5)
C10—Fe01—C1—C11	44.2 (2)	C3—Fe01—C7—C8	−75.5 (3)
C4—Fe01—C1—C11	156.73 (19)	C1—Fe01—C7—C6	81.5 (2)
C3—Fe01—C1—C11	−160.01 (19)	C5—Fe01—C7—C6	43.2 (4)
C5—C1—C2—C3	−0.1 (2)	C8—Fe01—C7—C6	−117.1 (3)
C11—C1—C2—C3	174.7 (2)	C2—Fe01—C7—C6	125.7 (2)
Fe01—C1—C2—C3	59.83 (16)	C9—Fe01—C7—C6	−79.6 (2)
C5—C1—C2—Fe01	−59.92 (15)	C10—Fe01—C7—C6	−36.69 (19)
C11—C1—C2—Fe01	114.9 (2)	C4—Fe01—C7—C6	−155.8 (4)
C7—Fe01—C2—C3	124.5 (2)	C3—Fe01—C7—C6	167.35 (18)
C1—Fe01—C2—C3	−119.5 (2)	C6—C7—C8—C9	0.9 (4)
C5—Fe01—C2—C3	−80.70 (16)	Fe01—C7—C8—C9	−60.0 (2)
C6—Fe01—C2—C3	166.91 (19)	C6—C7—C8—Fe01	61.0 (2)
C8—Fe01—C2—C3	81.0 (2)	C7—Fe01—C8—C9	118.6 (3)
C9—Fe01—C2—C3	45.6 (4)	C1—Fe01—C8—C9	164.0 (2)
C10—Fe01—C2—C3	−159.0 (4)	C5—Fe01—C8—C9	−38.0 (5)
C4—Fe01—C2—C3	−37.07 (15)	C6—Fe01—C8—C9	79.6 (2)
C7—Fe01—C2—C1	−115.9 (2)	C2—Fe01—C8—C9	−158.67 (18)
C5—Fe01—C2—C1	38.82 (13)	C10—Fe01—C8—C9	37.0 (2)
C6—Fe01—C2—C1	−73.6 (2)	C4—Fe01—C8—C9	−74.5 (2)
C8—Fe01—C2—C1	−159.45 (19)	C3—Fe01—C8—C9	−116.3 (2)
C9—Fe01—C2—C1	165.2 (3)	C1—Fe01—C8—C7	45.4 (4)
C10—Fe01—C2—C1	−39.5 (5)	C5—Fe01—C8—C7	−156.6 (3)
C4—Fe01—C2—C1	82.46 (14)	C6—Fe01—C8—C7	−39.0 (2)
C3—Fe01—C2—C1	119.5 (2)	C2—Fe01—C8—C7	82.8 (3)
C1—C2—C3—C4	0.1 (3)	C9—Fe01—C8—C7	−118.6 (3)
Fe01—C2—C3—C4	58.62 (17)	C10—Fe01—C8—C7	−81.6 (3)
C1—C2—C3—Fe01	−58.54 (15)	C4—Fe01—C8—C7	166.9 (2)
C7—Fe01—C3—C2	−76.1 (2)	C3—Fe01—C8—C7	125.1 (3)
C1—Fe01—C3—C2	38.08 (14)	C7—C8—C9—C10	−0.7 (4)
C5—Fe01—C3—C2	82.93 (15)	Fe01—C8—C9—C10	−59.9 (2)
C6—Fe01—C3—C2	−43.0 (5)	C7—C8—C9—Fe01	59.2 (2)
C8—Fe01—C3—C2	−118.1 (2)	C7—Fe01—C9—C10	81.1 (3)
C9—Fe01—C3—C2	−160.32 (17)	C1—Fe01—C9—C10	−41.8 (5)
C10—Fe01—C3—C2	166.0 (2)	C5—Fe01—C9—C10	−75.1 (2)
C4—Fe01—C3—C2	120.0 (2)	C6—Fe01—C9—C10	36.5 (2)
C7—Fe01—C3—C4	163.9 (2)	C8—Fe01—C9—C10	119.5 (3)
C1—Fe01—C3—C4	−81.92 (15)	C2—Fe01—C9—C10	169.2 (3)
C5—Fe01—C3—C4	−37.07 (14)	C4—Fe01—C9—C10	−116.6 (2)
C6—Fe01—C3—C4	−163.0 (4)	C3—Fe01—C9—C10	−158.2 (2)
C8—Fe01—C3—C4	121.9 (2)	C7—Fe01—C9—C8	−38.4 (3)
C2—Fe01—C3—C4	−120.0 (2)	C1—Fe01—C9—C8	−161.3 (3)
C9—Fe01—C3—C4	79.7 (2)	C5—Fe01—C9—C8	165.5 (2)
C10—Fe01—C3—C4	46.0 (3)	C6—Fe01—C9—C8	−83.0 (3)
C2—C3—C4—C5	0.0 (3)	C2—Fe01—C9—C8	49.7 (4)
Fe01—C3—C4—C5	58.38 (17)	C10—Fe01—C9—C8	−119.5 (3)
C2—C3—C4—Fe01	−58.41 (17)	C4—Fe01—C9—C8	124.0 (2)
C7—Fe01—C4—C5	−168.4 (4)	C3—Fe01—C9—C8	82.3 (3)
C1—Fe01—C4—C5	−38.69 (13)	C7—C6—C10—C9	0.4 (3)
C6—Fe01—C4—C5	49.5 (3)	Fe01—C6—C10—C9	59.4 (2)
C8—Fe01—C4—C5	161.79 (17)	C7—C6—C10—Fe01	−59.0 (2)
C2—Fe01—C4—C5	−83.30 (15)	C8—C9—C10—C6	0.2 (4)
C9—Fe01—C4—C5	121.45 (16)	Fe01—C9—C10—C6	−59.4 (2)
C10—Fe01—C4—C5	80.33 (17)	C8—C9—C10—Fe01	59.6 (2)
C3—Fe01—C4—C5	−120.4 (2)	C7—Fe01—C10—C6	38.3 (2)
C7—Fe01—C4—C3	−48.0 (5)	C1—Fe01—C10—C6	−75.3 (2)
C1—Fe01—C4—C3	81.69 (15)	C5—Fe01—C10—C6	−117.3 (2)
C5—Fe01—C4—C3	120.4 (2)	C8—Fe01—C10—C6	82.8 (3)
C6—Fe01—C4—C3	169.9 (2)	C2—Fe01—C10—C6	−44.5 (5)
C8—Fe01—C4—C3	−77.8 (2)	C9—Fe01—C10—C6	120.2 (3)
C2—Fe01—C4—C3	37.08 (14)	C4—Fe01—C10—C6	−159.2 (2)
C9—Fe01—C4—C3	−118.17 (17)	C3—Fe01—C10—C6	168.4 (3)
C10—Fe01—C4—C3	−159.29 (15)	C7—Fe01—C10—C9	−81.9 (3)
C3—C4—C5—C1	0.0 (3)	C1—Fe01—C10—C9	164.5 (2)
Fe01—C4—C5—C1	59.18 (15)	C5—Fe01—C10—C9	122.6 (2)
C3—C4—C5—Fe01	−59.20 (17)	C6—Fe01—C10—C9	−120.2 (3)
C2—C1—C5—C4	0.1 (2)	C8—Fe01—C10—C9	−37.4 (2)
C11—C1—C5—C4	−174.96 (19)	C2—Fe01—C10—C9	−164.7 (4)
Fe01—C1—C5—C4	−60.18 (16)	C4—Fe01—C10—C9	80.6 (2)
C2—C1—C5—Fe01	60.26 (15)	C3—Fe01—C10—C9	48.2 (4)
C11—C1—C5—Fe01	−114.8 (2)	C2—C1—C11—O1	−177.9 (2)
C7—Fe01—C5—C4	171.4 (3)	C5—C1—C11—O1	−3.9 (3)
C1—Fe01—C5—C4	118.6 (2)	Fe01—C1—C11—O1	−89.6 (2)
C6—Fe01—C5—C4	−157.43 (17)	C2—C1—C11—C12	4.3 (3)
C8—Fe01—C5—C4	−48.9 (4)	C5—C1—C11—C12	178.23 (19)
C2—Fe01—C5—C4	80.04 (16)	Fe01—C1—C11—C12	92.5 (2)
C9—Fe01—C5—C4	−77.10 (19)	O1—C11—C12—C13	13.0 (3)
C10—Fe01—C5—C4	−117.04 (17)	C1—C11—C12—C13	−169.09 (17)
C3—Fe01—C5—C4	36.79 (15)	C14—N1—C13—C12	70.6 (3)
C7—Fe01—C5—C1	52.8 (4)	C11—C12—C13—N1	72.3 (2)
C6—Fe01—C5—C1	83.95 (18)	C13—N1—C14—C15	19.6 (3)
C8—Fe01—C5—C1	−167.5 (3)	C13—N1—C14—C19	−163.0 (2)
C2—Fe01—C5—C1	−38.59 (13)	N1—C14—C15—C16	175.5 (2)
C9—Fe01—C5—C1	164.27 (14)	C19—C14—C15—C16	−1.9 (4)
C10—Fe01—C5—C1	124.33 (15)	C14—C15—C16—C17	0.6 (5)
C4—Fe01—C5—C1	−118.6 (2)	C15—C16—C17—C18	1.1 (5)
C3—Fe01—C5—C1	−81.84 (14)	C16—C17—C18—C19	−1.3 (5)
C7—Fe01—C6—C10	−119.1 (3)	C17—C18—C19—C14	−0.1 (4)
C1—Fe01—C6—C10	123.40 (19)	N1—C14—C19—C18	−175.8 (2)
C5—Fe01—C6—C10	79.4 (2)	C15—C14—C19—C18	1.7 (4)
C8—Fe01—C6—C10	−80.2 (2)		

### Hydrogen-bond geometry (Å, °)

**Table:**

*D*—H···*A*	*D*—H	H···*A*	*D*···*A*	*D*—H···*A*
N1—H1N···O1^i^	0.80 (3)	2.30 (3)	3.082 (3)	164 (2)
C19—H19···O1^i^	0.93	2.65	3.425 (3)	141
C4—H4···N1^ii^	0.93	2.63	3.489 (3)	153

Symmetry codes: (i) −*x*+1, −*y*, −*z*+1; (ii) *x*−1, *y*+1, *z*.
